# A Self-Regulation–Based eHealth and mHealth Intervention for an Active Lifestyle in Adults With Type 2 Diabetes: Protocol for a Randomized Controlled Trial

**DOI:** 10.2196/12413

**Published:** 2019-03-22

**Authors:** Louise Poppe, Ilse De Bourdeaudhuij, Maïté Verloigne, Laurent Degroote, Samyah Shadid, Geert Crombez

**Affiliations:** 1 Physical Activity and Health Research Group Department of Movement and Sports Sciences Ghent University Ghent Belgium; 2 Ghent Health Psychology Lab Department of Experimental Clinical and Health Psychology Ghent University Ghent Belgium; 3 Department of Endocrinology Ghent University Hospital Ghent Belgium

**Keywords:** protocol, randomized controlled trial, eHealth, mHealth, type 2 diabetes, self-regulation, physical activity, sedentary behaviour, mobile phone

## Abstract

**Background:**

Adoption of an active lifestyle plays an important role in the management of type 2 diabetes. Online interventions targeting lifestyle changes in adults with type 2 diabetes have provided mixed results. Previous research highlights the importance of creating theory-based interventions adapted to the population’s specific needs. The online intervention “MyPlan 2.0” targets physical activity and sedentary behavior in adults with type 2 diabetes. This intervention is grounded in the self-regulation framework and, by incorporating the feedback of users with type 2 diabetes, iteratively adapted to its target population.

**Objective:**

The aim of this paper is to thoroughly describe “MyPlan 2.0” and the study protocol that will be used to test the effectiveness of this intervention to alter patients’ levels of physical activity and sedentary behavior.

**Methods:**

A two-arm superiority randomized controlled trial will be performed. Physical activity and sedentary behavior will be measured using accelerometers and questionnaires. Furthermore, using questionnaires and diaries, patients’ stressors and personal determinants for change will be explored in depth. To evaluate the primary outcomes of the intervention, multilevel analyses will be conducted.

**Results:**

The randomized controlled trial started in January 2018. As participants can start at different moments, we aim to finish all testing by July 2019.

**Conclusions:**

This study will increase our understanding about whether and how a theory-based online intervention can help adults with type 2 diabetes increase their level of physical activity and decrease their sedentary time.

**International Registered Report Identifier (IRRID):**

DERR1-10.2196/12413

## Introduction

Diabetes is associated with various health problems including kidney failure, retinopathy, and cardiovascular disease [1]. By 2035, it is estimated that one in ten adults will have diabetes [1]. This exponential growth of diabetes is largely accounted for by type 2 diabetes, which is responsible for 85%-95% of the disease cases [1]. Adopting an active lifestyle (ie, being physically active and limiting sedentary behavior) has shown to play an important role in both the prevention and management of type 2 diabetes [2,3]. Consequently, cost-effective approaches that help adults with type 2 diabetes in increasing their physical activity and reducing their sedentary behavior are needed.

Electronic health (eHealth) and mobile health (mHealth) interventions have the potential to reach large populations in a cost-effective way and are effective in promoting an active lifestyle in the general population [4]. Nevertheless, research about the effectiveness of online interventions targeting adults with type 2 diabetes reveals mixed results [5-7]. Based on these findings, several proposals have been formulated to better design and implement eHealth and mHealth interventions for adults with type 2 diabetes. First, interventions should be grounded in and informed by theoretical models [5,7,8]. Research revealed that online programs that are developed using theoretical models result in larger effect sizes [9]. A useful perspective may well be the self-regulation framework, which focuses on both preintentional (such as increasing knowledge) and postintentional (such as action and coping planning) processes of behavior change [10]. This framework describes behavior change as a goal-guidance process starting from personal determinants for change until goal maintenance or, if necessary, disengagement [11]. Second, online interventions should take into account the perspective and needs of the users. This can be accomplished by involving end users during the entire developmental process of the online program [12,13]. Third, developers should address the high levels of attrition that are negatively affecting many online interventions [14]. Combining a website with a reminder system, such as automated emails or text messages, may be one of the ways to reinforce website use [7].

There are many papers discussing the effects of online interventions. Nevertheless, a clear and thorough description of the interventions themselves is often missing. This impedes research, as researchers often start from scratch when creating an intervention. The publication of study protocols that clearly describe the active ingredients and the “dose” of the interventions are therefore needed [5]. This study describes the protocol for a randomized controlled trial examining how a self-regulation–based eHealth and mHealth intervention (“MyPlan 2.0”) targeting sedentary behavior and physical activity influences the behavior-change process of adults with type 2 diabetes. The needs of adults with type 2 diabetes were taken into account, as they were actively involved in the development of the program [15,16]. “MyPlan 2.0” consists of a website that motivates users to create, follow, and maintain their own goals for physical activity or sedentary behavior in combination with an optional mobile app offering daily support. The aim of this paper is to describe “MyPlan 2.0” and provide the study protocol that will be used to investigate the website’s effectiveness and underlying mechanisms. The items addressed in this protocol paper are based on the 2013 Standard Protocol Items: Recommendations for Interventional Trials (SPIRIT) statement [17]. Multimedia Appendix 1 presents the completed SPIRIT checklist.

## Methods

### Ethical Approval

This study was approved by the Committee of Medical Ethics of the Ghent University Hospital (Belgian registration number: B670201732566) and registered as a clinical trial (Clinicaltrials.gov NCT03291171). Written informed consent from each participant will be obtained. Precautions will be taken to ensure participants’ privacy during data analysis.

### Study Design

**Figure 1:**
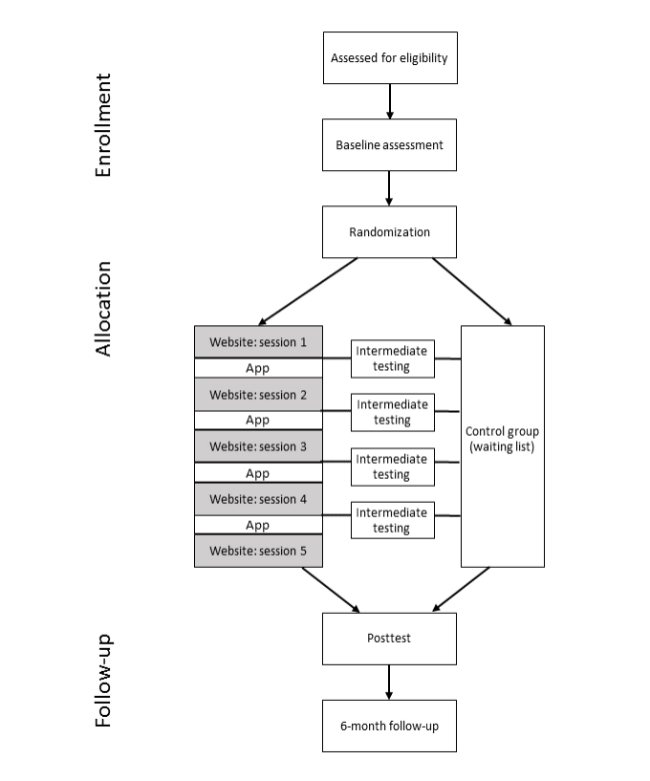
Study flow.

A two-arm superiority randomized controlled trial will be performed. The study flow is depicted in Figure 1. Data will be collected during three home visits. During the first home visit, written informed consent will be obtained from the participants, and the participants will be asked whether they would like to increase their physical activity or decrease their sitting time. Participants will then complete questionnaires on physical activity, sedentary behavior, personal determinants for change (eg, self-efficacy), and health-related outcomes. Furthermore, participants’ weight and waist circumference will be assessed. Finally, participants from both groups will wear an accelerometer for a period of 10 days and fill out a morning and evening diary on each of these days. The diaries will assess participants’ daily goals and possible person-related barriers (ie, fatigue, stress, depressed mood, pain, nausea, and feelings of numbness or tingling in limbs).

After this period, LP will randomly allocate participants to the waiting list control group or the intervention group in a 1:2 allocation ratio by using an automated randomizer [18]. This will be done independent from patients’ choice to increase their physical activity or decrease their sitting time. Participants allocated to the intervention group who chose to increase their physical activity will be directed to the website targeting physical activity, whereas participants who chose to decrease their sitting times will be directed to the website targeting sedentary behavior. Participants owning a smartphone will be asked to download the mobile app. The website part of the intervention consists of five consecutive modules (a start module and four follow-up modules) spread over a 5-week period. Each week, participants from both groups will be phoned by a researcher. During these phone calls, questions regarding participants’ personal determinants for behavior change (eg, self-efficacy) will be repeated. In doing so, we will achieve the temporal separation needed to investigate causal pathways [19]. Furthermore, the phone calls will be used to check whether patients had hypoglycemia or made changes to their medication.

One week after completing the program (for the intervention group) or 6 weeks after finishing the baseline measures (for the control group), a second home visit will be scheduled during which the posttest will be carried out. In this phase, questions regarding process evaluation will be added to the questionnaires of the intervention group. Finally, 6 months after the baseline test, the intervention group will be visited a third time by the researchers to perform the follow-up test in order to examine whether the potential effects of the intervention are sustainable.

### Hypotheses

Our primary hypotheses for this study are as follows: (1) The intervention group allocated to the module “Physical Activity” will show an increase in total physical activity from pre- to posttest compared to no change in the control group. This effect will be sustained in the intervention group from the posttest to the follow-up test. (2) The intervention group allocated to the module “Sedentary Behaviour” will show a decrease in sedentary behavior from pre- to posttest compared to no change in the control group. This effect will be sustained in the intervention group from the posttest to the follow-up test.

Our secondary hypotheses are as follows: (1) Positive changes in physical activity or sedentary behavior will be mediated by increases in the personal determinants self-efficacy, action planning, and coping planning. (2) The intervention group will have more positive health outcomes (ie, a lower weight; smaller waist circumference; and lower levels of fatigue, anxiety, and depression) from pre to follow-up test. (3) The negative effect of daily stressors (ie, fatigue, stress, depressed mood, pain, nausea, and feelings of numbness or tingling in limbs) on physical activity and sedentary behavior will be smaller in the intervention group from pre- to posttest compared to no change in the control group. This effect will be sustained in the intervention group from posttest to follow-up test.

As moderation analyses for online interventions targeting adults with type 2 diabetes are not usually performed [5], no hypotheses regarding the moderation effects are made. The following factors will be examined as potential moderators: age, sex, education, and chosen behavior (ie, physical activity or sedentary behavior).

### Participants

The required sample size was calculated using the software GPower 3.1.9.2 [20]. This program requires the following input: effect size, alpha, power, number of groups, and number of measurements. To our knowledge, there is no meta-analysis documenting the effect sizes of online interventions targeting physical activity or sedentary behavior in adults with type 2 diabetes. As people with type 2 diabetes tend to be overweight and physically inactive, we decided to focus on these characteristics for our effect-size estimation [21]. A meta-analysis by Davies et al (2012) showed that eHealth interventions targeting physical activity levels of overweight or sedentary adults reached effect sizes of 0.37 [22]. Most of the studies included in the meta-analysis used questionnaires rather than accelerometers to measure participants’ level of physical activity. Assuming an effect size of 0.37, alpha of .05, beta of .90, two groups (intervention group and control group), and three measurements (pretest, posttest, and follow-up test), the *a priori* power analysis suggests a sample size of 96 (64 participants in the intervention group and 32 in the control group).

Therefore, 96 participants with type 2 diabetes will be recruited via the Ghent University Hospital, the Sint-Lucas General Hospital (Ghent), the Maria Middelares General Hospital (Ghent), and the Damiaan General Hospital (Ostend). To be eligible for participation, participants should have type 2 diabetes, have been diagnosed for at least 1 month, be 18 years or older, speak Dutch, be computer literate, have internet access, and not have participated in previous studies on “MyPlan 2.0.” Participants receiving concomitant care and interventions will not be excluded. Potential participants with type 2 diabetes will be recruited via the endocrinologists of the collaborating hospitals. The endocrinologists will check whether visiting patients meet the inclusion criteria, provide eligible patients with a flyer, and ask these patients if the researchers are allowed to contact them. If the patient agrees, the researchers will receive the patient’s contact details. The recruitment procedures will continue until the proposed number of participants is reached. Except during the pretest, neither the participants nor the researchers assessing the outcome variables will be blinded.

### Description of the Intervention

“MyPlan 2.0” is an eHealth and mHealth intervention targeting physical activity and sedentary behavior. The program is based on “MyPlan 1.0,” a self-regulation-based eHealth intervention (ie, a website) originally designed to be used by general practitioners in order to increase the levels of physical activity and the intake of fruit and vegetables in the general population [23]. Although “MyPlan 1.0” was shown to be effective, the high levels of attrition indicated that there was room for improvement [24-27]. Moreover, the general practitioners indicated that the program should also be made available to people with type 2 diabetes, as health self-regulation is of great importance in this population [28]. For “MyPlan 2.0,” we decided to focus on physical activity and sedentary behavior. Two studies were performed to guide the adaptations to the program. First, user and website characteristics related to attrition were explored [29]. Second, think-aloud interviews were performed with 20 adults with type 2 diabetes and 20 adults from the general population [15]. We instructed users to verbalize their thoughts while using “MyPlan 1.0.” Based on the findings of both studies, a new version—“MyPlan 2.0 version T2D”—was developed. Using semistructured interviews with 21 adults with type 2 diabetes who had completed “MyPlan 2.0,” this version was further adapted to users with type 2 diabetes [16].

“MyPlan 2.0” consists of a website and a mobile app. The website, created using LifeGuide [30], is the basis of the intervention and has five consecutive parts. The first time a user logs into the website, (s)he can choose whether (s)he would like to be more physically active or less sedentary. The further structure of the website is independent of the chosen health behavior. First, in order to provide tailored feedback and personalized information (eg, the age and sex of the persons in the success stories are tailored to the user’s age and sex), all users answer questions assessing demographic information. Subsequently, users have the option to take a quiz regarding the benefits of the selected health behavior. Next, users fill in a questionnaire to assess their current levels of physical activity or sedentary behavior and receive feedback regarding the time they spend being physically active or sitting. Thereafter, users create a specific plan for increasing their physical activity (eg, “On Monday morning I will walk 10 minutes in the neighbourhood”) or decreasing their sedentary behavior (eg, “I will stand when talking on the phone”). Users will then state possible barriers for the selected goal, search for solutions, and decide how they will monitor their goal. Offered choices are a calendar, a booklet, the mobile app, etc. Next, users will see an overview of their goals, barriers, and solutions and how they will monitor their behavior change: This is called the action plan. Finally, users will be offered additional information about how they can receive social support from their environment.

The intervention lasts for 5 weeks. Each week, the users receive an email to go back to the website to evaluate and adapt their goal based on the successes and failures of the past week. In these follow-up sessions (four in total), users can actively reflect on their behavioral change. Each follow-up session has the same structure. First, users see the goal(s) they have set the week before and are asked whether they reached their goal. Feedback based on success or failure is given. Second, users choose to keep or adapt their goal. Third, users think about possible barriers that might come up in the following week and search for solutions. Fourth, users see an overview of their (new) goal, barriers, and solutions. Fifth, users can read additional tips and tricks to be more physically active or less sedentary. Table 1 gives an overview of the behavior change techniques that are covered by the website. The techniques are labelled according to the taxonomy of behavior change techniques compiled by Michie and colleagues [31].

The mobile app offers daily support during the entire behavior change process. Through the app, users can review their goals, monitor their progression, search for possible coping techniques, and take quizzes regarding physical activity or sedentary behavior. By visiting the website, completing quizzes, and monitoring their behavior change, users can collect points in the mobile app. This gaming element was added to increase engagement with the intervention. The techniques implemented in the mobile app can be found in Table 2. The techniques are labelled according to the taxonomy of behavior change techniques compiled by Michie and colleagues [31]. Multimedia Appendix 2 presents screenshots from the website and the mobile app.

### Measurement instruments

#### Questionnaires

##### Demographic Variables

Participants’ age, sex, height, civil status, education, profession, and the time since diagnosis will be assessed using a questionnaire in the pretest.

##### Physical Activity and Sedentary Behavior

The Dutch version of the long International Physical Activity Questionnaire (IPAQ-L) [32] and the Longitudinal Aging Study Amsterdam (LASA) sedentary behavior questionnaire [33] will be used to assess the context-specific physical activity and sedentary behavior. The interview version of the IPAQ-L and the LASA questionnaires will be conducted, as previous research showed that participants tend to overreport their levels of physical activity when using self-administered questionnaires [34]. This will be done during each of the three testing waves.

##### Health Outcomes

Participants’ feelings of depression, anxiety, and fatigue will be assessed during each testing wave using scales of the Patient-Reported Outcomes Measurement Information System [35]. Feelings of depression and anxiety will be measured via the Dutch version of the depression short-form scale (version 1.0) and anxiety short-form scale (version 1.0), both of which contain six items with five answer options: “never,” “seldom,” “sometimes,” “often,” and “always.” Participants’ fatigue will be measured using the subscale “fatigue” of the Dutch version of the 29-profile scale (version 2.01). The subscale contains four items with five answer options: “not at all,” “a bit,” “somewhat,” “to a fairly high degree,” and “to a high degree.”

##### Personal Determinants

Personal determinants for behavior change (ie, self-efficacy, risk perceptions, outcome expectations, motivation, intention, action planning, coping planning, and self-monitoring) will be measured in both groups during each testing wave and the weekly phone calls. These determinants will be assessed using multiple items (minimum three items per determinant) that were selected by presenting a large number of items measuring Health Action Process Approach (HAPA) determinants to 11 experts in the self-regulation framework. All experts indicated whether each item measured the presented HAPA determinant and how sure they were of their answer [36]. Based on these responses, discriminant content validity was assessed using the method described by Johnston et al [36], and the best scoring items were selected. Each item has 10 answer options, ranging from “completely disagree” to “completely agree.”

#### Accelerometry

Participants’ sedentary time and total, moderate-to-vigorous, and light physical activity will be assessed for a period of 10 days during each of the three testing waves using ActiGraph accelerometers (model GT3X+; Pensacola, FL), which have been shown to be reliable and valid [37-40].

#### Anthropometry

Anthropometry will be carried out on each of the three testing waves (ie, during each home visit). The visiting researcher will assess participants’ weight using a Seca weighing scale (model 813; Benson Avenue, CA), whereas waist circumference will be measured at the lowest rib margin and the iliac crest at the midaxillary line using Seca measuring tape.

#### Diary

##### Mental and Physical Well-Being

Each morning and evening, participants will rate the extent of fatigue, stress, depressed mood, pain, nausea, and feelings of numbness or tingling in the limbs experienced by using a 10-point scale, ranging from “absolutely not” to “very much.”

##### Action Planning

Each morning, participants will report their planned actions for that day by indicating which type of goals they planned (eg, social activities, work, and physical activity). Each evening, participants will report the level to which they reached their listed goals by using a 10-point scale, ranging from “did not work out” to “worked out very well.” An overview of the measures and the time points during which they will be assessed is shown in Table 3.

**Table 1:** Overview of the self-regulation techniques implemented in the website.

Self-regulation technique	Implementation mode
Providing information on the consequences of behavior, in general	During session 1, users have the option of taking a quiz. The quiz contains questions regarding the benefits of the chosen health behavior (ie, increasing physical activity or reducing sedentary behavior). Each answer is followed by a clear explanation.
Exploring social support	During session 1, users can read more information about how they can obtain social support from their partner, friends, family, or colleagues.
Providing feedback on performance	During session 1, users complete a short questionnaire regarding their current levels of physical activity or sedentary behavior. Thereafter, they can see for how much time they are physically active or sedentary and in which domains (eg, transport or leisure time).
Action planning	In each session, users have the option to create their own goals to increase their physical activity or decrease their sedentary behavior. By answering different questions, the goals are made as specific as possible (eg, “On Monday and Wednesday morning I will walk 10 minutes in the neighbourhood”).
Barrier identification/problem solving	In each session, users are prompted to think about possible barriers regarding their plans and search for potential solutions (eg, “I might forget my plan to take a walk in the evening, so I will stick a note on the fridge”).
Prompting self-monitoring of behavior	In each session, the website encourages users to monitor their behavior change and presents options to do so.
Prompting review of behavioral goals	During each follow-up session, users are asked to review the extent to which the goals set in the previous session were achieved.

**Table 2:** Overview of the self-regulation techniques implemented in the mobile app.

Self-regulation technique	Implementation mode
Providing information on the consequences of behavior, in general	Users have the option to take several quizzes on the benefits of the chosen health behavior (ie, increasing physical activity or reducing sedentary behavior).
Prompting self-monitoring of behavior	Every evening, users receive a notification to fill in whether they were more active today than they used to be before. The entries of each week are shown in a graph visible to the user.
Action planning	Users can review their goals and make adaptations, if necessary. In the mornings of days during which users should live up to their goal, a notification is sent to remind them about the goal.
Barrier identification/problem solving	Users can see an overview of common barriers and solutions for these barriers.

Cognitive interviews, usually performed in small samples [41], were used to assure the comprehensibility of the diary and questionnaire assessing personal determinants for behavioral change [42,43]. We purposively selected participants aged ≥50 years, because the prevalence of type 2 diabetes peaks in older age [1]. The participants were instructed to read and complete the diary and questionnaire. For each item, the interviewer (LP) asked the participant whether (s)he considered the item to be difficult, how (s)he came to an answer, and which time period (s)he took into account when providing an answer. Based on the results of these interviews, adaptations to the items were made. The mean (SD) age of the participants was 58.3 (6.5) years (range, 52-67 years). Demographic information of the participants is provided in Table 4.

#### Data Quality Assurance

The data-collection process will be guided and monitored by the researchers. As this study is part of a postgraduate doctoral degree project, no specific data trial steering or data monitoring committee was assigned. However, the study progress will be discussed monthly with the research team. Only accelerometer data from participants who had 4 valid days including 1 weekend day (“valid” defined as ≥10 hours of wear time) will be included in the analysis [44]. Furthermore, responses to the IPAQ-L and LASA questionnaires will be checked for plausibility. For the IPAQ, we will use the method described by Dubuy et al [45] to truncate the data. For the LASA questionnaire, we will truncate the data to a maximum total score of 16 hours a day [46].

#### Statistical Analysis

Statistical analysis will be performed after completing the data-collection phase. No interim analysis will be executed. Descriptive statistics and independent samples *t* tests will be carried out to explore and identify potential differences between the intervention and the waiting-list control group. To evaluate the primary outcomes of the intervention, three-level (hospital, patient, and time) analyses will be conducted. Intention-to-treat analyses will be performed. As the drop-out rate is usually high in eHealth research [14], it is likely that a per protocol analysis will not be feasible. Furthermore, participants of the intervention group will only be included in the analysis if they complete four of five sessions on the website. Moderating effects will be identified via interaction terms (including the possible moderator). For the secondary outcomes, mediating effects will be investigated using structural equation modelling. Changes in health outcomes and the effect of daily stressors on patients’ activity levels will be assessed using multilevel analysis. Data analysts will not be blinded to participants’ group allocation.

**Table 3:** Overview of the measures.

Measures		Baseline	Intermediate test	Posttest	Follow-up test
Demographic information using the general questionnaire		✓			
**Physical activity and sedentary behavior**					
	Accelerometer	✓		✓	✓
	IPAQ-L^a^	✓		✓	✓
	LASA^b^ sedentary behavior questionnaire	✓		✓	✓
**Health outcomes**					
	Weight	✓		✓	✓
	Waist circumference	✓		✓	✓
	PROMIS^c^ fatigue	✓		✓	✓
	PROMIS depression	✓		✓	✓
	PROMIS anxiety	✓		✓	✓
Personal determinants - single items		✓	✓	✓	✓
**Daily stressors and goals**					
	Fatigue	✓		✓	✓
	Stress	✓		✓	✓
	Feelings of depression	✓		✓	✓
	Pain	✓		✓	✓
	Nausea	✓		✓	✓
	Numbness/tingling in limbs	✓		✓	✓
	Goals	✓		✓	✓
	Evaluation of goals	✓		✓	✓

^a^IPAQ-L: long International Physical Activity Questionnaire.

^b^LASA: Longitudinal Aging Study Amsterdam.

^c^PROMIS: Patient-Reported Outcomes Measurement Information System.

**Table 4:** Demographic information of the participants from the cognitive interviews (N=4).

Demographics		N
Women		3
**Level of education**		
	Primary school	1
	Secondary education	1
	College	2
Diagnosed with type 2 diabetes		2

### Process Evaluation

#### Contextual Factors

Individuals live in certain contexts that inevitably shape their lifestyle. As the design of the environment plays an important role in developing and maintaining an active way of living [47], patients’ perception of the environment will be examined during the pretest. This will be done via the short version of the Assessing Levels of Physical Activity questionnaire, which has shown to be valid and reliable [48]. Furthermore, we will check for physical conditions that may have hindered the participant from being active. This will be examined during the posttest and the follow-up tests using the question, “In the past six weeks, were there physical factors (e.g. sickness or injury) making it hard for you to be physically active?” In case the participants give a positive answer, they will be asked to describe the physical factor.

**Overview of the questions assessing participants’ satisfaction with the website and the mobile app.**

**Satisfaction with the website (scale: 1 - very poor to 10 - outstanding):**

1. Overall, to what extent did you like the website of ‘MyPlan 2.0’?
2. To what extent did you like the quiz?
3. To what extent did you like the questionnaire and the accompanying feedback?
4. To what extent did you like the action planning module?
5. To what extent did you like the coping planning module?
6. To what extent did you like the tips and tricks section?
7. To what extent did you like the feedback in the follow-up sessions?

**Satisfaction with the mobile app (scale: 1 - very poor to 10 - outstanding):**

1. Overall, to what extent did you like the mobile application of ‘MyPlan 2.0’?
2. To what extent did you like the quizzes?
3. To what extent did you like the monitoring module?
4. To what extent did you like the action planning module?
5. To what extent did you like the coping planning module?
6. To what extent did you like the points collection module?

**Satisfaction with “MyPlan 2.0” as a whole (scale: 1 - not at all to 5 - very much):**

1. Was the information and support delivered by ‘MyPlan 2.0’ comprehensible ?
2. Was the information and support delivered by ‘MyPlan 2.0’ useful?
3. Was the information and support delivered by ‘MyPlan 2.0’ personally relevant to you?
4. Was the information and support delivered by ‘MyPlan 2.0’ motivating?
5. Did you enjoy using ‘MyPlan 2.0’?

#### Usage of the Website and the Mobile App

LifeGuide allows researchers to monitor website usage and time spent on the website. Participants from the intervention group who do not return to the website after receiving the reminder email will be contacted by phone by one of the researchers. The time point and number of these calls will be monitored for each participant.

#### Satisfaction With the Website and the Mobile App

Users’ satisfaction with both the website and the mobile app will be assessed using questionnaires during the posttest and by analyzing the usage data. Textbox 1 gives an overview of the questionnaire items and response categories. The questions are based on items used in other studies examining the appreciation of online interventions [49,50]. Participants who did not use the mobile app will not receive the questions regarding appreciation of the mobile app. Time spent on the website and the number of optional pages visited will be assessed by analyzing the website usage data.

### Dropout

To gain insight into participants’ reasons for attrition, several questions will be asked in case participants decide to quit using the program. Textbox 2 gives an overview of the questions and their accompanying scale. These questions are created by the research team based on a viewpoint article regarding attrition in eHealth by Eysenbach [14].

**Overview of the questions about participants’ reasons for attrition. Scale for all questions was 1 (not at all) to 5 (very much), except question number 17 (response options: yes/no).**

1. ‘MyPlan 2.0’ lived up to my expectations.
2. The website of ‘MyPlan 2.0’ is userfriendly.
3. The mobile application of ‘MyPlan 2.0’ is userfriendly.
4. My diabetes educator reacted positively regarding my participation in ‘MyPlan 2.0’.
5. My GP reacted positively regarding my participation in ‘MyPlan 2.0’.
6. My friends and family reacted positively regarding my participation in ‘MyPlan 2.0’.
7. ‘MyPlan 2.0’ helped me to be more physically active/to sit less.
8. The personal contact with the researchers of ‘MyPlan 2.0’ were an additional reason for me to participate.
9. Going through ‘MyPlan 2.0’ took a lot of my time.
10. Filling out the questionnaires took a lot of my time.
11. I did not like wearing the accelerometer.
12. I did not like being weighed and measured.
13. I doubted to participate in this study.
14. While taking part in the study drastic changes in my life occurred (e.g. death of a family member, had a (grand)child, new job, etc.).
15. I can work well with a computer.
16. When I have computer problems, I can rely on others to help me.
17. I also took part in other programmes targeting a healthy way of living.

### Informed Consent

All participants will be required to provide written informed consent before starting the study (ie, during the first home visit). Each participant will be informed about the design of the study, its purpose, confidentiality of data, and the fact that (s)he has the right to leave the study at any time without stating any reason.

### Adverse Effects

Adverse effects are defined as negative outcomes related to participation in the study. Possible adverse effects in this study might be injury or severe hypoglycemia resulting from increased physical activity. The occurrence of adverse effects will be recorded and evaluated for both the intervention and control groups.

### Data Storage

All data will be stored on a password-protected computer and central disk space. Data from the website will additionally be stored on password-encrypted servers. Only persons who are part of the research team will have access to the data. Multimedia Appendix 3 presents the data-management plan.

### Incentives

To encourage participants to fill out their diaries, draw lots will be given based on the number of questions answered. The intervention group and the waiting-list control group will have equal chances to win prizes (ie, gift vouchers of popular supermarkets).

## Results

Development of the website and the mobile app is complete. The randomized controlled trial started in January 2018. As participants can start the study at different times, we aim to complete all testing by July 2019. Important protocol modifications will be reported on Clinicaltrials.gov. The results of the study will be communicated via publications. For these publications, the American Psychological Association guidelines for authorship eligibility will be followed.

## Discussion

### Overview

Adopting an active lifestyle is key in the management of type 2 diabetes [3]. As the prevalence of adults with type 2 diabetes is increasing [1], self-management interventions that can be applied to large groups are welcomed. Online interventions have the possibility to reach many users at the same time and have shown to be effective in altering health behaviors, especially when they are theory based [4,9]. “MyPlan 2.0” is a theory-based website and mobile app for motivating and supporting adults with type 2 diabetes to be more physically active and less sedentary.

### Study Implications

This study will test the effectiveness of “MyPlan 2.0” for each phase of the behavior change process using a randomized controlled trial. More specifically, this trial will investigate whether the program can increase patients’ physical activity and decrease their sitting time. Furthermore, we will determine whether these potential changes are mediated by alterations in personal determinants for change and result in positive health outcomes. Through the diaries, we will gain more insight into patients’ daily struggles to adopt an active way of living. Finally, potential differences based on participants’ characteristics will be explored. Consequently, the implications of this study will contribute to the literature of both the theoretical and practical domain of eHealth and mHealth, targeting self-management in adults with type 2 diabetes.

This study design has several limitations. First, as the resources for this study are limited, we will not be able to collect a large sample size. Consequently, it might be more difficult to identify statistically significant intervention effects. This issue highlights the importance of preventing dropout from the intervention. Dropout will be prevented by sending reminders to participants who are not logging in for follow-up sessions on the website via emails and phone calls. Second, considering the important role of creating a feeling of “goal-ownership” in self-regulation theory, participants can freely choose between the components increasing physical activity and decreasing sedentary behavior. We can therefore not ensure that the two components will have the same number of users. As a result, it might be more difficult to detect an effect for sedentary behavior if a large group selects physical activity as their target behavior and vice versa. As the structure of the intervention and the implemented behavior change techniques are exactly the same for both target behaviors, we decided to perform the analysis with one, rather than two, intervention groups. However, the selected behavior will be added as a moderator to the analysis. Third, in order to test our hypotheses, participants will need to fill out many questionnaires. This might cause higher levels of attrition. Fourth, participants are called weekly by the researchers to check for hypoglycemia or alterations in medication and to assess participants’ personal determinants for change via an interview. Due to these weekly phone calls, participants might show higher levels of engagement with the intervention than they normally would. However, as we will also implement these weekly calls in the control group, we believe that the calls will have a limited impact on the intervention effects. Finally, as the researcher who will analyze the data will also be involved in the data-collection process, blinding of the data analyst is not possible. To account for this issue, a strict protocol has been developed for processing and analyzing the data.

## Appendix

Multimedia Appendix 1 Completed SPIRIT checklist. SPIRIT: Standard Protocol Items: Recommendations for Interventional Trials.

Multimedia Appendix 2 Screenshots from the website and the mobile app.

Multimedia Appendix 3 Data management file.

## Abbreviations

eHealth: electronic health
HAPA: Health Action Process Approach
IPAQ-L: long International Physical Activity Questionnaire
LASA: Longitudinal Aging Study Amsterdam
mHealth: mobile health
PROMIS: Patient-Reported Outcomes Measurement Information System
SPIRIT: Standard Protocol Items: Recommendations for Interventional Trials
