# Supplementary material for: A Self-Regulation–Based eHealth and mHealth Intervention for an Active Lifestyle in Adults With Type 2 Diabetes: Protocol for a Randomized Controlled Trial
Source: JMIR Res Protoc. 2019 Mar 22;8(3):e12413. doi: 10.2196/12413 (PMC6450483; doi:10.2196/12413)
Supplement: Multimedia Appendix 2 [file resprot_v8i3e12413_app2.pdf]

MyPlan 2.0 - Screenshots

<u>Screenshots from the website:</u>

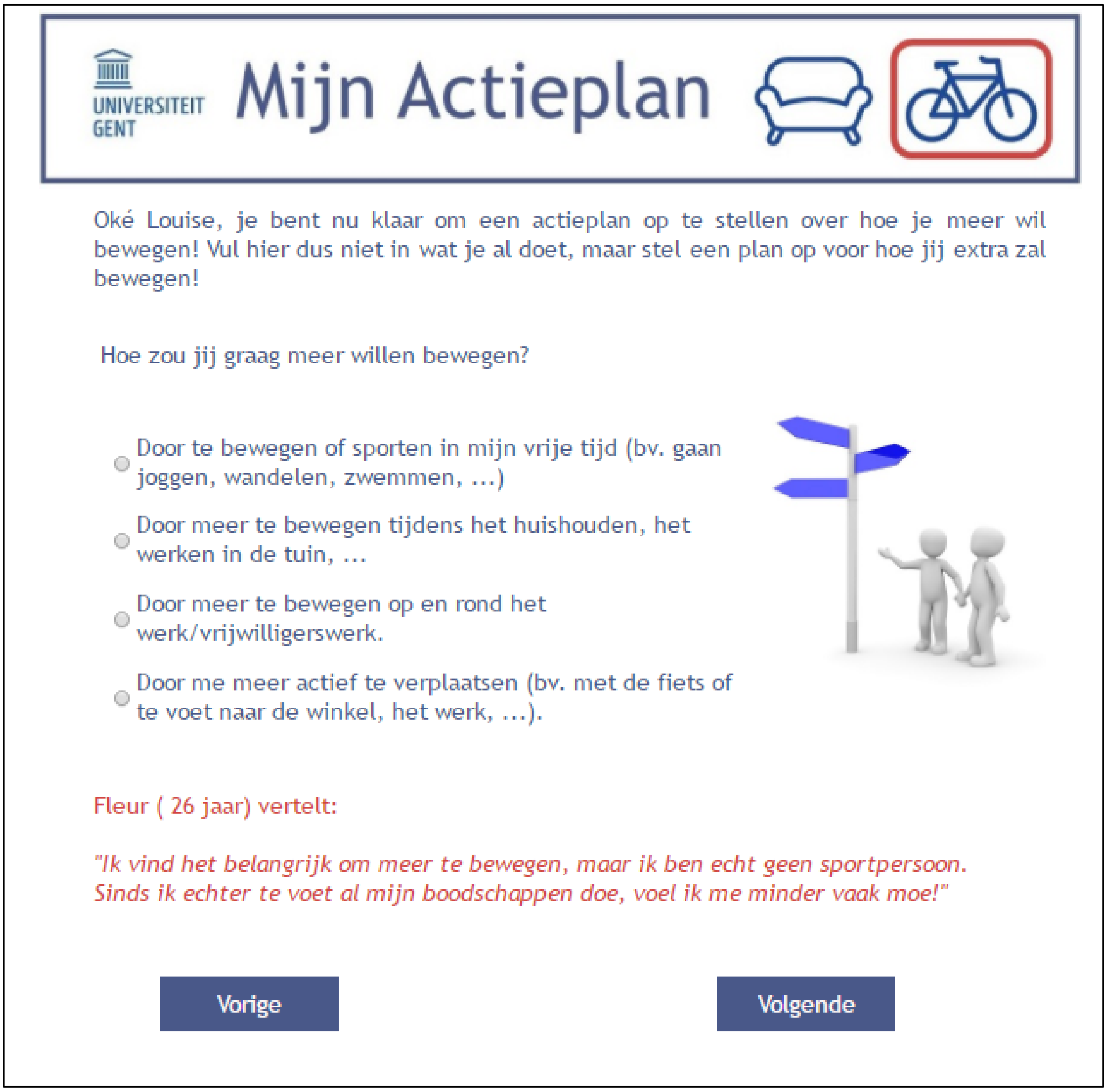

Figure 1. Defining an action in the action planning module

The text in red is a success story.

Translation: “Fleur (26 years old) tells: I consider it important to be more physically active, but I am not a sporty person. Since I do all my groceries by foot, I feel less tired during the day!”.

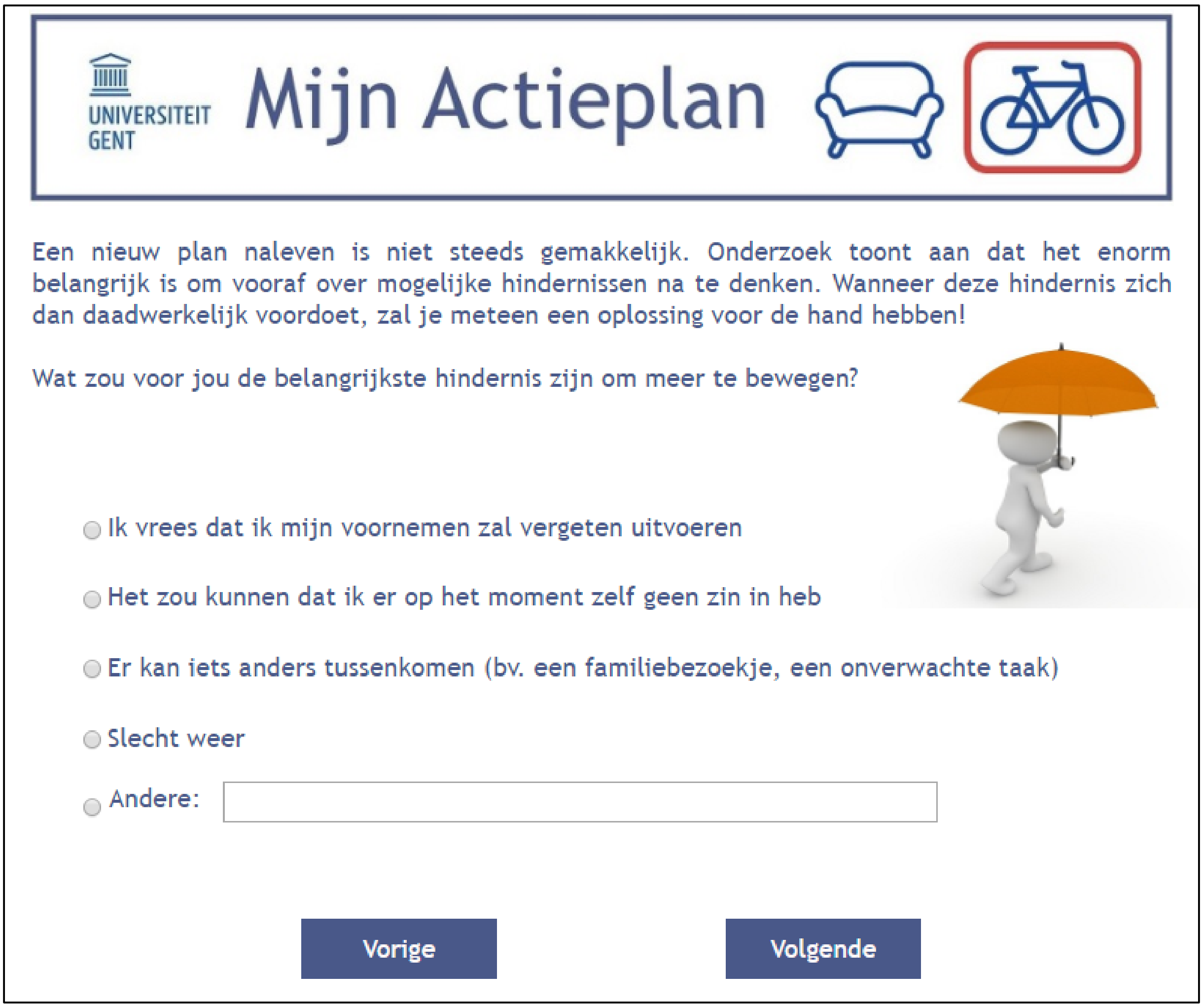

Figure 2. The coping planning module

Translation: “Living up to a new plan is not always easy. Research shows that it is really important to consider potential hindrances in advance. When this hindrance appears, you will have an immediate solution at hand!”

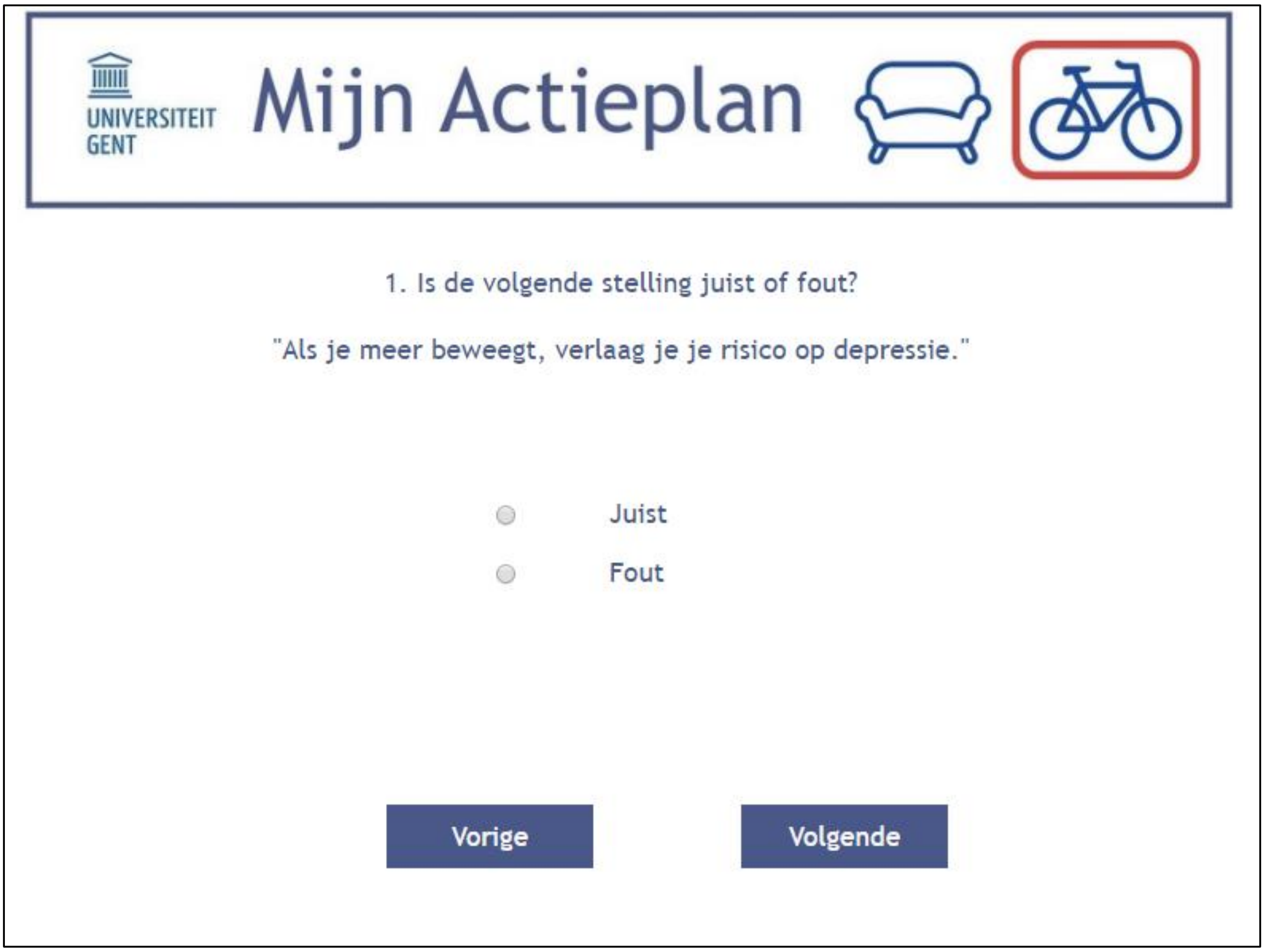

Figure 3. Example of a quiz question

Translation: “Is the following statement true or false? I you are more physically active, you have a lower chance on developing a depression.”

Screenshots from the mobile application:

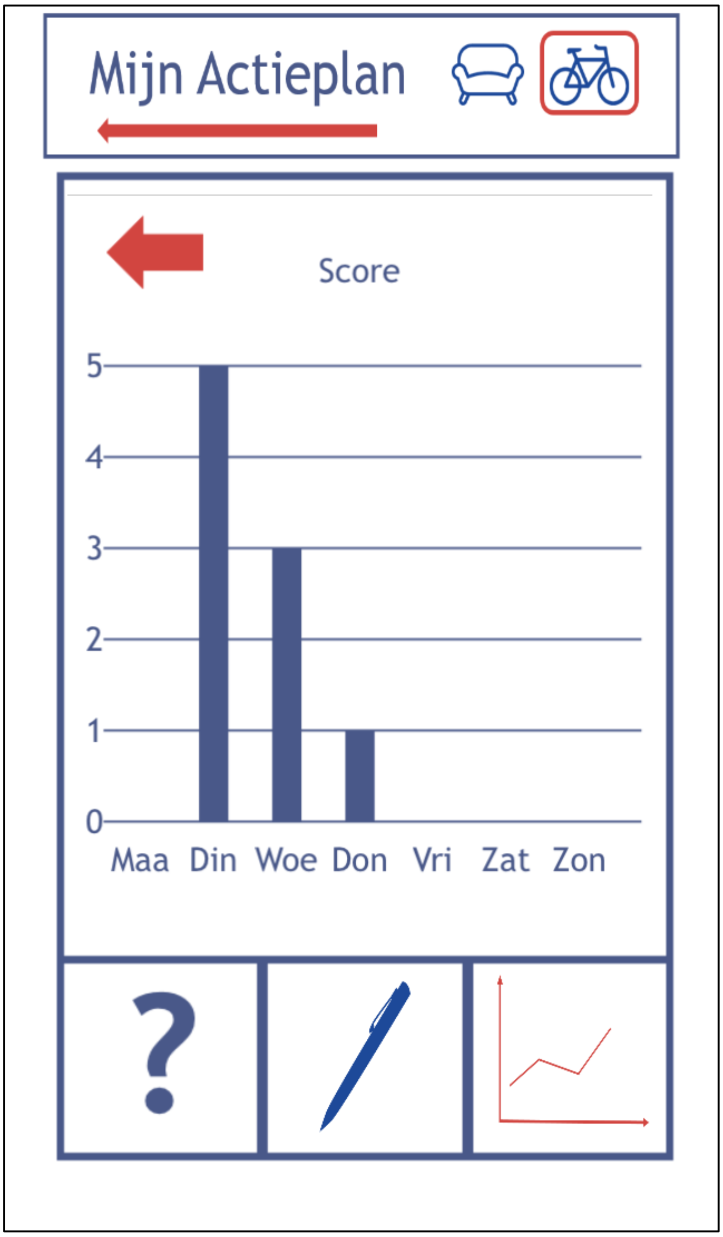

Figure 4. Monitoring health behaviours

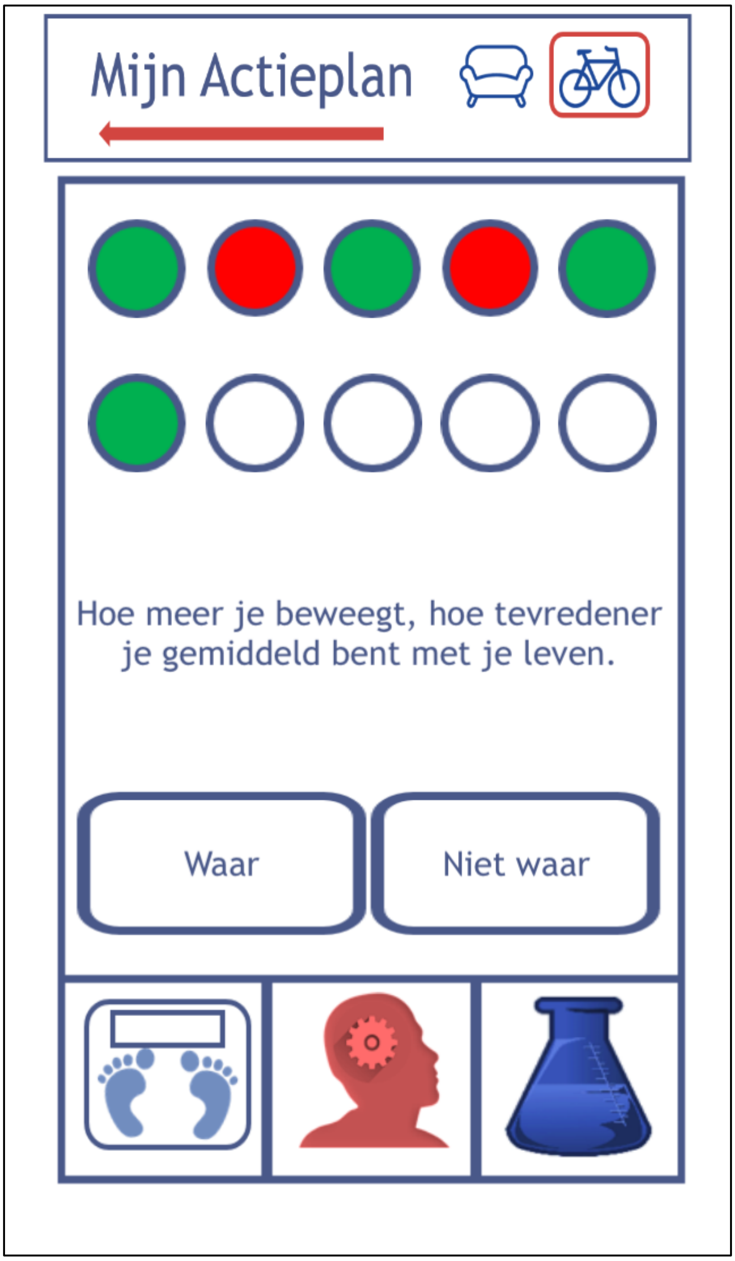

Figure 5. Quizzes

Translation: “In general, the more people are physically active, the more happy they are with their lives.” Blue boxes: “True” and “False”.

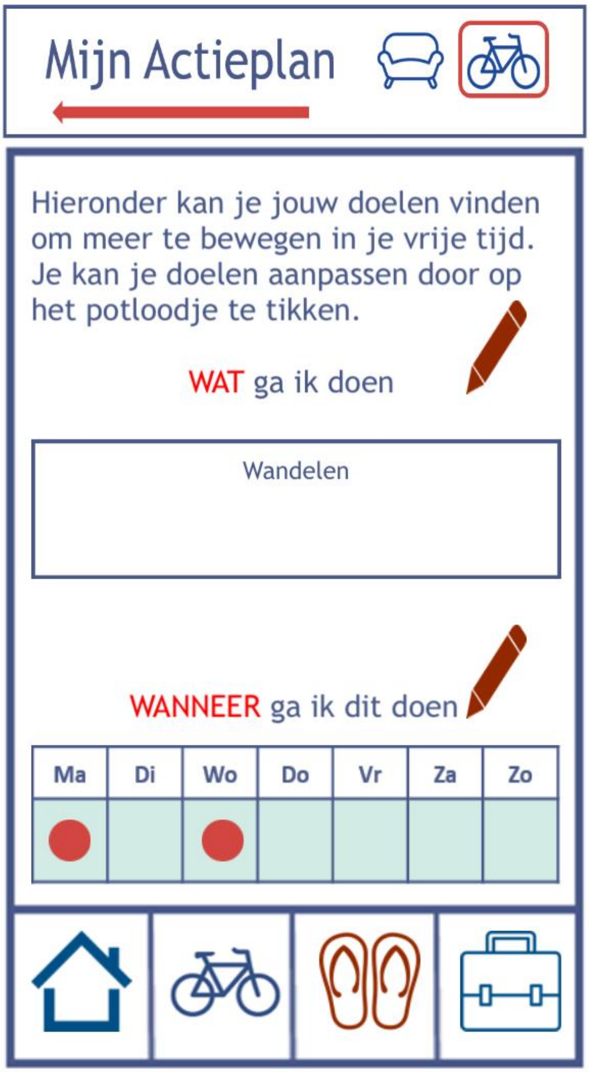

Figure 6. Revising and adapting plans

Translation: “Below you can find your goals to be more physically active during leisure time. You can adapt your goals by tapping on the pencil icon.”

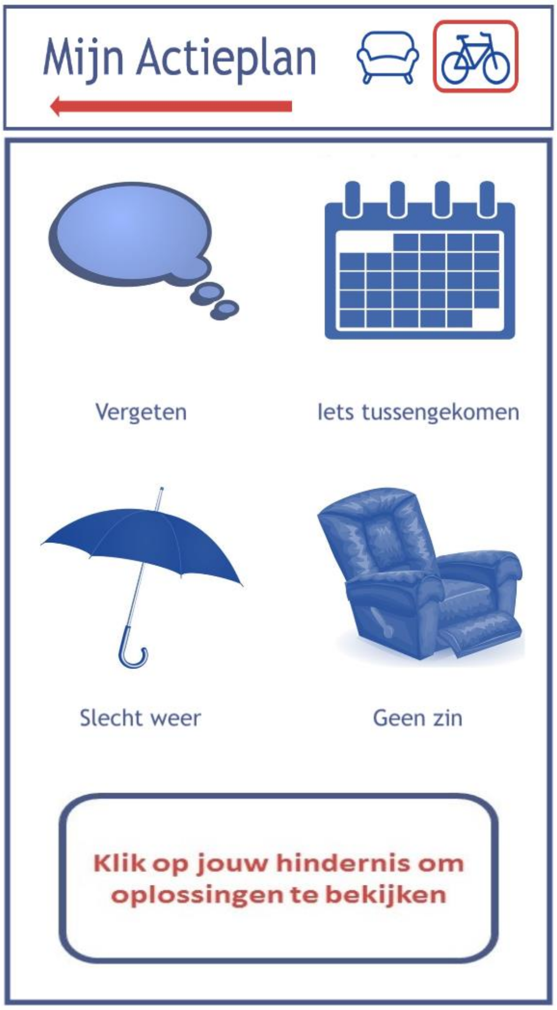

Figure 7. Coping planning module

Translation: “Tap on a hindrance to find solutions”.
