# Supplementary material for: A Self-Regulation–Based eHealth and mHealth Intervention for an Active Lifestyle in Adults With Type 2 Diabetes: Protocol for a Randomized Controlled Trial
Source: JMIR Res Protoc. 2019 Mar 22;8(3):e12413. doi: 10.2196/12413 (PMC6450483; doi:10.2196/12413)
Supplement: Multimedia Appendix 3 [file resprot_v8i3e12413_app3.pdf]

Data Management Plan

1 Information about the data that will be collected or used

1.1 Description of the data that will be collected

A randomised controlled trial will be executed to test the effectiveness of 'MyPlan 2.0' to alter the levels of physical activity and sedentary behaviour of adults with type 2 diabetes. The data will be collected before, during and after the intervention. Furthermore, to investigate long term effects, a final testing phase will be performed six months after the first testing phase. We will use questionnaires to asses self-reported physical activity, sedentary behaviour and participants' psychosocial determinants for change (e.g. attitude, intention, etc.), in combination with motion sensors to asses objective physical activity/sedentary behaviour. Furthermore, via the use of diaries patients' daily stressors and goals to adopt an active lifestyle will be investigated. Participants' weight and waist circumference will be assessed via a weighting scale and a tape measure. Finally, other health outcomes such as lowered levels of fatigue will be assessed via questionnaires.

1.2 File formats that will be used

Website data (collected via LifeGuide software) and data from the mobile application will be extracted in Excel files and stored in one SPSS file. The physical activity and sedentary behaviour data will be retrieved from the accelerometers using ActiLife software. The format of the data will be '.AGD'. The SPSS file will be completed with the essence of these data. The diary data will be added to the same SPSS file. All data collected during each of three testing waves will be stored in three SPSS documents, one document per measurement time. After finalization of the data collection, data will be grouped in one SPSS file. Statistical analysis will be performed using R Studio.

1.3 Documentation of the data

- **Study level documentation:**

All contextual information, such as background, information about the research design, context of data collection, etc. will be documented in a study protocol, that will be updated regularly.

- **Data level documentation:**

An excel file inventory of the datasets will be made to list all the datasets and to document the relationships between the different datasets. Names of the SPSS files, containing the different datasets, will also be documented in this inventory.
Variable labels will be defined and stored in SPSS itself, where the data is stored and analyzed. All information about the variables included in these datasets will also be gathered in an excel codebook. The codebook will contain variable names, labels, codes, classification, abbreviations, item information, missing data codes, etc.
The steps that were taken to structure and analyze these data will be documented in a logbook.

1.4 Risks and potential difficulties during data collection and processing

It is important to have enough data to conduct the analyses. To avoid drop out users not logging in for following sessions will be phoned by one of the researchers. To make sure that the measures are reliable and valid, we will make use of validated motion sensors and questionnaires. All data collected will be checked for missing values (e.g. a missing day in the motion sensor data) and loss of information.

1.5 Data storage

To prevent loss, all data will be stored at the provided central storage infrastructure (“central share”) of Ghent University, where the data are secure. Once retrieved, data will be deleted from recording devices and surveyservers. Questionnaires will be stored in the foreseen cupboards in the department or in the archive of the faculty after scanning them and thus making the information digitally available.

1.6 Back-up of the data

The central share of Ghent University, where all data will be stored, provides daily automated back-ups. On the share "snapshots" of the data are made. Snapshots allow you to retrieve (older versions of) files. To retrieve previous versions you can go back 15 weeks in total, based on 5 weeks of daily snapshots and 15 weeks of weekly snapshots.

1.7 Data security?

All cupboards (that store questionnaires and informed consents) are locked and can only be accessed by the researchers from our research group. All computers are provided with personal passwords and are weekly scanned for malware. Digital files will be coded, using participant codes instead of names. Only the researcher and the promotors will have access to a file containing the links between the codes and the personal data of the participants (name, address, etc.). All data will be stored at the central storage infrastructure of Ghent University and are therefore automatically protected. When destroying files after digitalizing them, we will make sure this is done properly by a shredder. Paper documents can also be collected and destroyed at university level.

1.8 Data access

All researchers from our research team (two PhD students, one post-doctoral researcher and two promotors) will have access to the data. During this study master students will be involved in the data collection and will get access to (part of) the data as well. Therefore they will sign a declaration of confidentiality.

2 Ethical Issues

2.1 Data collection, storage, processing and archiving

Participants will be informed on every aspect of the study that concerns their participation (e.g. data collection and storage, anonymization, etc.) and give written informed consent for their participation. All the data will be stored and processed confidentially, in accordance with the Belgian Law of 8 December 1992 on the protection of privacy in relation to the processing of personal data, the Belgian law of 22 August 2002 on the rights of patients, and the European regulation of 14 April 2016 on data

protection. This study was approved by the Committee of Medical Ethics of the Ghent University hospital (Belgian registration number: B670201732566) and registered as clinical trial on https://register.clinicaltrials.gov (ID number: NCT03291171).

2.2 Data sharing

All participants receive an information letter in which ethical and privacy consequences will be explained. Data will only be accessible to the persons described above. If (parts of) datasets are shared with other researchers, all shared data will be anonymized. Only the researcher and the promotors (and research assistants when they recruited the participants) will have the rights to link the data to the participants.
